# Polychlorinated Biphenyls in the Plasma and Preen Oil of Black-Footed Albatross (*Diomedea nigripes*) Chicks and Adults on Midway Atoll, North Pacific Ocean

**DOI:** 10.1371/journal.pone.0123041

**Published:** 2015-04-22

**Authors:** Jun Wang, Sarah A. L. Caccamise, Lee Ann Woodward, Qing X. Li

**Affiliations:** 1 Department of Molecular Biosciences and Bioengineering, University of Hawaii at Manoa, Honolulu, Hawaii, 96822, United States of America; 2 U. S. Fish and Wildlife Service, Pacific Reefs NWRC, Honolulu, Hawaii, 96850, United States of America; The University of Iowa, UNITED STATES

## Abstract

Polychlorinated biphenyls (PCBs) are ubiquitous in the environment. Midway Atoll, located in the North Pacific Ocean, was occupied by the military during and after World War II. However, Midway Atoll has become a national wildlife refuge and home to many different seabirds today, including the black-footed albatross (*Diomedea nigripes*) (BFAL). The profiles and toxic equivalents (TEQ) of PCB congeners in the plasma and preen oil of BFAL chicks and adults were determined in this study. The concentrations of the total PCBs in the plasma samples of chicks and adults collected in Midway Atoll ranged from 2.3 to 223.8 (mean 80.1) and 22.8 to 504.5 (mean 158.6) ng g^-1^ (wet weight, ww), respectively. The TEQs ranged from 0.2 to 0.6 (mean 0.4) and 0.4 to 1.6 (mean 0.9) pg g^-1^ ww, respectively, in the plasma samples of chicks and adults from Midway Atoll. The major congeners in the plasma samples of chicks and adults included PCBs 31, 87, 97, 99, 118, 138, 153, and 180, accounting for 70% of the total PCBs. The concentrations of the total PCBs in the adult preen oil samples ranged from 1693 to 39404 (mean 10122) ng g^-1^ (ww), of which 97% were PCBs 105, 118, 128, 138, 153, 161, 172, and 183.

## Introduction

Midway Atoll is located in the North Pacific Ocean, approximately 1100 miles northwest of Oahu, Hawaii (approximately 177°W longitude, 28°N latitude). It is made up of two main islands (Sand Island and East Island) that are surrounded by a coral reef. It played a historical role in World War II and was used and modified during and after the war. Many industrial products including polychlorinated biphenyls (PCBs) were heavily used during military occupation. It is now part of the Hawaiian Islands National Wildlife Refuge and since May 1996 has been managed by the U.S. Fish and Wildlife Service. Midway Atoll and the waters surrounding it are home to millions of seabirds including black-footed albatrosses (BFAL) (*Phoebastria nigripes*). Albatross populations in the tropical North Pacific Ocean decreased due to hunting in the early 1900’s [1]. The population of BFAL has been steadily increasing because of prohibition of hunting and improvement of the marine environment [1]. The BFAL, however, is still considered a near threatened species because of potential losses to longline of fishing fleets, sea-level rise and storm surges (http://www.iucnredlist.org/details/22698350/0, accessed November 22, 2014).

PCBs are ubiquitous in the environment. PCBs are synthetic chemical mixtures and were used as additives in dielectric fluids in capacitors, transformers, and etc. [2,3]. The distribution of PCB congeners differs among trophic levels largely due to natural weathering, bioaccumulation, bioconcentration, and metabolism [4,5]. Therefore, the PCB congener distributions in an organism differ greatly from the congener distribution of the original technical mixture [4,5].

Several recent studies [6–8] were focused on persistent organic pollutants (POPs) in different environmental media of Midway Atoll. Those studies suggested local PCB pollution sources on Tern Island, French Frigate Shoals of the North Pacific Ocean. However, it is not clear whether the PCBs present in Midway Atoll are the result of local contamination, global distribution of pollutants, or a combination of both.

To measure the relative toxicities of persistent organic pollutants including PCBs, a toxic equivalency factor (TEF) scheme and other schemes have been developed and well accepted in the literature [9,10]. The toxicities are compared and rated against that of the most toxic congener 2,3,7,8-terachlorodibenzo-*p*-dioxin (TCDD) [9,10]. Twenty of the 209 PCB congeners have one and non-*ortho* chlorine substituents, and these congeners can form a planar structure similar to that of TCDD and 2,3,7,8-tetrachlorodibenzofuran (TCDF). As the number of ortho chlorines increases, the planarity of the congener decreases [11–13]. PCBs 77, 126, and 169 contain 4, 5, and 6 chlorine atoms in the non-*ortho* positions, respectively, with a putative mode of toxicity similar to TCDD. In the Great Lakes fish-eating birds, PCBs have a greater contribution to the toxic equivalents (TEQ) than polychlorinated dibenzodioxins (PCDDs) or polychlorinated dibenzofurans (PCDFs) [14,15]. Non-*ortho* PCB congeners in tissues of the organism are more toxic and higher bioaccumulation potential than other PCB congeners and these mixtures of non-*ortho* PCB congeners are also more toxic than the original Aroclor [9–14].

To our knowledge, comprehensive studies have not been reported for the concentrations of PCB congeners in the plasma and preen oil samples collected from live BFAL chicks and adults. The interest in PCB congener specific analyses has arisen because PCBs have shown distinct bioaccumulation potential and toxic effects on humans and wildlife [9,10,15–17]. The objectives of this study were to (1) determine residual levels, compositions, and distribution of PCBs in the plasma and preen oil of BFAL chicks and adults on Midway Atoll, North Pacific Ocean and compare samples of plasma and preen oil of the individual birds, (2) determine the toxic equivalents in the plasma samples, and (3) elucidate possible routes of PCB exposure to BFAL on Midway Atoll.

## Materials and Methods

### Sample information

**Fig 1:**
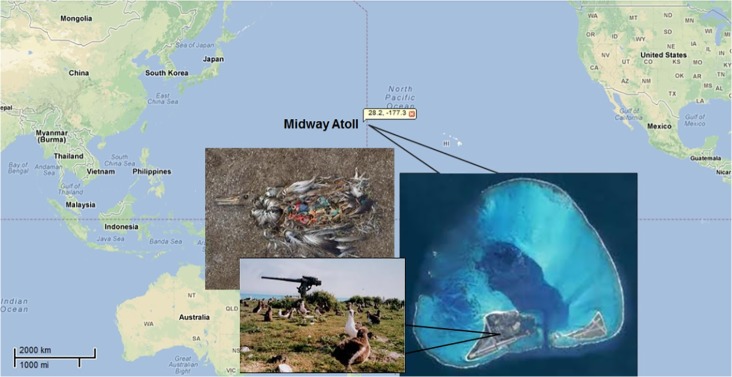
Locations of study area in Midway Atoll, North Pacific Ocean.

The U.S. Fish and Wildlife Service (USFWS) personnel under appropriate permits collected 22 chick and adult BFAL samples from the B8 and bulky dump sampling sites in Midway Atoll, North Pacific Ocean on May 8–10, 2001 (Table 1). The map of sampling sites was presented in Fig 1. Two groups of adult birds were chosen to collect samples. One group was comprised of 11 adult birds in the incubation period, while another group consisted of 11 adult birds in the early chick rearing period with chicks varying in age between 7 and 12 days. The body conditions of the latter group were expected to be poorer than that of the former, due to reproductive stress. The age of 22 chicks ranged from 1 month to 3 months. The 22 adults and 22 chicks sampled were not matched pairs. The use of the plasma and preen oil from the birds allows for non-invasive sample collection. The BFAL blood samples (10 ml) were collected with the 20-ml syringe and placed in 20-ml centrifuge tubes prior to adding anticoagulant to prevent blood solidification. The blood samples were then centrifuged at 7100 × *g* for 10 min to obtain plasma. Preen oil samples were collected from preen gland via 1-ml syringe and placed in 5-ml glass bottles. All plasma and preen oil samples were stored at -20°C until preparation for analysis.

**Table 1:** Information of sampling sites, and chicks and adults bird samples collected in Midway Atoll.

**Albatross Chicks**			**Albatross Adults**			**Sampling Date**
**No.**	**BM** ^†^ **(kg)**	**Sampling Site**	**No.**	**BM** ^†^ **(kg)**	**Sampling Site**	
1	3.85	Bulky Dump Site	1	2.87	Bulky Dump Site	May 8, 2001
2	3.10	Bulky Dump Site	2	2.15	Bulky Dump Site	May 8, 2001
3	4.65	Bulky Dump Site	3	3.45	Bulky Dump Site	May 9, 2001
4	4.40	Bulky Dump Site	4	2.25	Bulky Dump Site	May 8, 2001
5	3.90	Bulky Dump Site	5	2.50	Bulky Dump Site	May 8, 2001
6	2.60	Bulky Dump Site	6	3.35	Bulky Dump Site	May 8, 2001
7	2.75	Bulky Dump Site	7	2.65	Bulky Dump Site	May 8, 2001
8	4.45	Bulky Dump Site	8	3.05	Bulky Dump Site	May 8, 2001
9	3.55	Bulky Dump Site	9	2.47	Bulky Dump Site	May 8, 2001
10	3.15	Bulky Dump Site	10	2.55	Bulky Dump Site	May 9, 2001
11	3.55	Bulky Dump Site	11	3.10	Bulky Dump Site	May 10, 2001
12	4.10	B8 Site	12	3.65	B8 Site	May 9, 2001
13	3.25	B8 Site	13	4.05	B8 Site	May 9, 2001
14	2.75	B8 Site	14	3.92	B8 Site	May 9, 2001
15	3.65	B8 Site	15	3.95	B8 Site	May 9, 2001
16	2.5	B8 Site	16	3.85	B8 Site	May 9, 2001
17	2.53	B8 Site	17	4.35	B8 Site	May 9, 2001
18	2.60	B8 Site	18	4.10	B8 Site	May 9, 2001
19	3.05	B8 Site	19	2.15	B8 Site	May 9, 2001
20	2.45	B8 Site	20	2.60	B8 Site	May 9, 2001
21	2.20	B8 Site	21	3.70	B8 Site	May 9, 2001
22	2.90	B8 Site	22	3.27	B8 Site	May 9, 2001

^†^ BM, body mass

### Extraction and cleanup

The plasma samples were extracted on a Dionex accelerated solvent extractor (ASE) 200 (Sunnyvale, CA). The plasma samples (1–2 g) were treated with methanol (1–2 ml) to lyse the red blood cells prior to extraction and were allowed to sit overnight in a chemical hood. The samples were then mixed with anhydrous sodium sulfate with a plasma/anhydrous sodium sulfate (w/w) ratio of 1/6 for the ASE extraction. The plasma samples were extracted with a hexane-acetone mixture (1:1, v/v) at a pressure of 1500 psi and temperature of 100°C. Two static cycles of 20 min each were performed. The extracts were dried with anhydrous Na_2_SO_4_. The extracts were reduced in volume to approximately 1 ml, followed by evaporation to near dryness under a gentle stream of nitrogen gas and further drying in a silica gel desiccator.

The dry extracts were dissolved with n-hexane and washed with 98% sulfuric acid to digest. The organic solvent was reduced to 1 ml, followed by column chromatographic cleanup. The column was composed of neutral silica gel (6 cm, 3% (w/w) deactivated), neutral alumina (10 cm, 3% (w/w) deactivated), and 1 cm of anhydrous sodium sulfate on the top, which was eluted with 30 ml of dichloromethane-hexane mixture (1:2 v/v) to yield the PCBs fraction. The fraction was concentrated to 20 μl under a gentle high purity nitrogen stream after 20 μl of isooctane was added as the trapping solvent. Each sample was analyzed in triplicate with a gas chromatograph-electron capture detector/mass spectrometer (GC-ECD/MS).The preen oil samples (1–8 mg) were manually extracted with hexane. The hexane extracts were then cleaned up with concentrated sulfuric acid, followed by column chromatography as discussed above for the plasma. The samples were then analyzed with GC-ECD/MS.

### Instrumental analysis

The samples were analyzed on a Varian Saturn 2000 GC-ECD/MS with simultaneous electron capture and mass spectrometric detection. The column flow was split between the ECD and the MS in a 1:10 ratio. The column used was a capillary column ZB-1 (60 m x 0.25 mm i.d. x 0.25 μm film; Phenomenex, Torrance, CA). Helium was used as the carrier gas, and nitrogen was used as the make-up gas for the ECD. The initial oven temperature was 120°C and was linearly ramped at 2°C min^-1^ from 120°C to 275°C (hold 10 min). The injector and ECD detector were set to 280°C and 320°C, respectively. The injection volume was 2 μl. The injection mode was splitless, and the purge time was 1.2 min. The temperature of the ion trap was 220°C, manifold 80°C, and the transfer line 210°C. The mass spectrometer was operated in the scan mode with a mass range 100 *m/z* to 650 *m/z*. The individual PCB congeners were identified with retention time correlation to PCB standards (AccuStandard, Inc., New Haven, CT) and mass spectral matches. PCB concentrations were calculated from external standards with ECD data and confirmed with the MS.

### Quality assurance and quality control

Mean PCB recoveries and relative standard deviations (RSDs) were first obtained to evaluate the method performance by multiple analyses of 10 replicates spiked BFAL plasma and preen oil samples with standard C-CS-08 (AccuStandard) that contained PCBs 30, 43, 55, 58, 76, 109, 112, 120, 159, 186, 192, and 198. The spike level of each PCB was 20 ng g^-1^ wet weight (ww). The BFAL plasma and preen oil samples in which PCBs were not detected were used for recovery tests. A solvent blank and matrix blank were analyzed through the entire procedure prior to and after every 10 samples. Working standard solutions of PCBs were run at the beginning of sample analysis to determine the relative response factors and evaluate peak resolution. Each sample was analyzed in triplicate unless otherwise stated. The limits of detection (LOD) were derived from the blanks and quantified as 3 times the standard deviation (SD) of the mean blank noises. In addition, peaks were only integrated when the signal-to-noise ratio was ≥3; otherwise, they were considered non-detected. The mean recoveries of different PCB congeners ranged from 95% to 121% with an mean RSD of 6.0% for plasma samples and from 84% to 118% with an average RSD of 8.2% for the preen oil samples. The LOD for individual PCBs ranged from 10 to 50 pg g^-1^. Reported PCBs concentrations were not corrected.

### Statistical analysis

Statistical analyses were performed with SAS (analysis of variance [ANOVA] SAS/STAT 6.03; SAS Institute, Carey, NC, USA). For all BFAL sampled twice in different sample periods, the mean concentrations were used in ANOVA. All data were presented as mean ± standard deviation. Correlations between log PCB concentrations and BFAL body weights were assessed with Microsoft Office Excel 2010.

## Results and Discussion

### PCB concentrations in the plasma of chicks and adults and toxicity assessment

**Fig 2:**
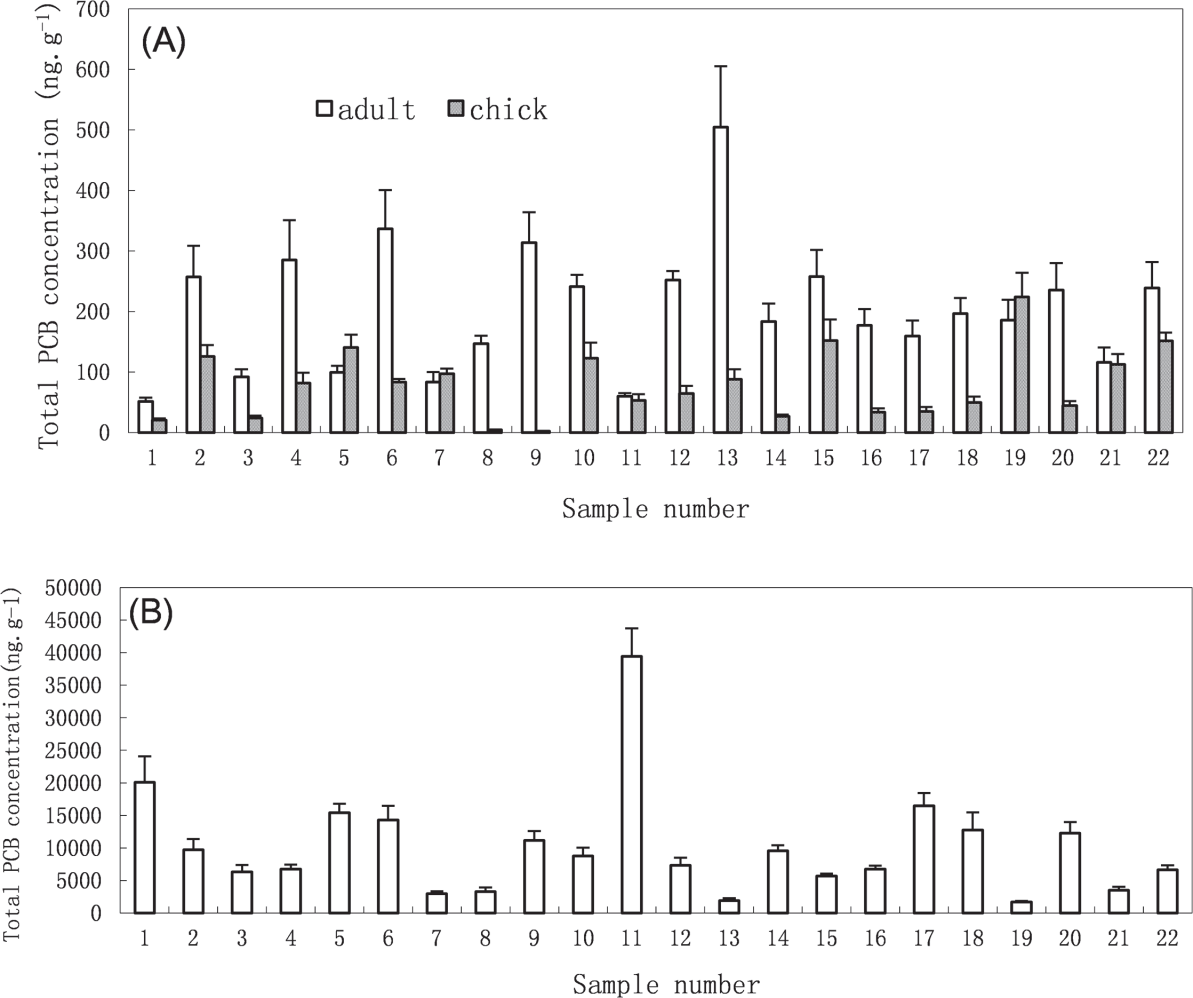
Total PCB concentration (ng g^-1^ ww) in the plasma (A) and preen oil (B) samples of BFAL chicks and adults from Midway Atoll, Northern Pacific Ocean.

**Fig 3:**
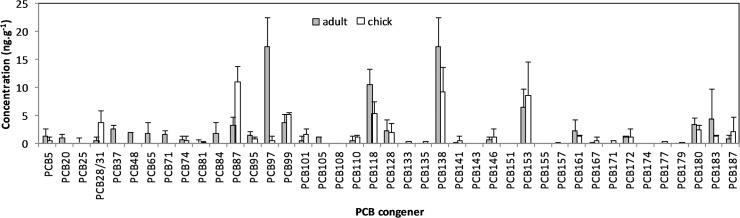
PCB congener distribution (ng g^-1^ ww) for plasma samples of BFAL from Midway Atoll, Northern Pacific Ocean.

**Fig 4:**
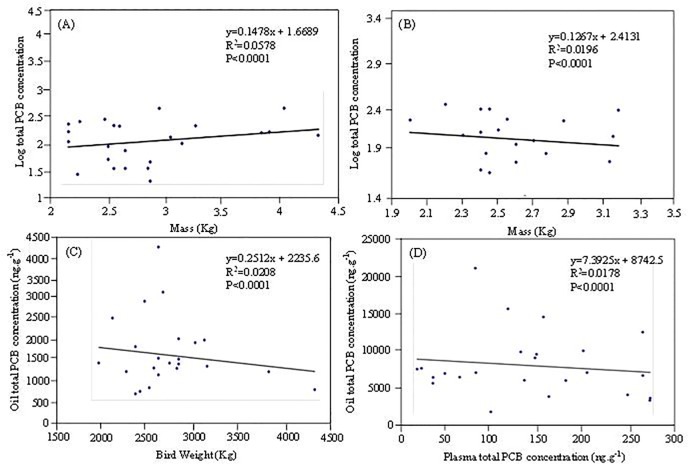
Scatter plots between total PCB concentration in the plasma samples and black-footed albatross adults (A) and chicks (B) body mass, between total PCB concentration in the preen oil samples and black-footed albatross adults body mass (C), and between total PCB concentration in the plasma and preen oil samples (D).

Fig 2A shows the concentrations of total PCBs in the plasma of chicks and adults. The concentrations of the total PCBs in the plasma of chick and adult samples collected in B8 and bulky dump sampling site ranged from 2.3 to 223.8 (mean 80.1) ng g^-1^ ww and 22.8 to 504.5 (mean 158.6) ng g^-1^ ww, respectively. PCB concentrations in most of these adult samples were higher than those of chick’s samples. The major congeners in the plasma of chick and adult samples included PCBs 31, 87, 97, 99, 118, 138, 153, and 180, accounting for 70% of the total PCBs (Fig 3). In most cases, the chicks had a higher body mass than the adults; especially in the bulky dump BFAL sampled, but did not necessarily contain the highest total PCB concentrations. Fig 4A and 4B present a group of scatter plots between the total PCB concentrations in the plasma samples and body masses of BFAL chicks and adults.

The total PCB concentrations in these BFAL samples were significantly (p < 0.0001) related to the total body masses. There was a significant negative correlation between body mass and total PCB concentration. Significant differences in concentrations of total PCB were observed among sampling periods (ANOVA; *p* < 0.0001). For BFAL adults, total PCBs were greatest throughout the entire nesting season. This may be due to mobilization of fat reserves at an increased rate due to extended periods of egg incubation without feeding and foraging over great distances to obtain food for the chicks.

TEQs were calculated for the plasma of chick and adult samples using TEFs for birds [9,10]. PCBs 81 and 126 have a TEF value of 0.1 in birds, while PCB 77 has a TEF of 0.05. PCBs 114 and 169 have a TEF value of 0.001 in birds. These five PCB congeners have the highest toxic effect on birds. The total TEQs ranged from 0.2 to 0.6 (mean 0.4) and 0.4 to 1.6 (mean 0.9) pg g^-1^ ww, respectively, in the plasma of chick and adult samples from two different nest sampling sites on Midway Atoll, North Pacific Ocean. All plasma samples contained PCBs 99, 118, 138, and 153, while PCB 138 was the most abundant PCB congener in all plasma samples. PCB 126 was not found in any chick and adult plasma samples. It is noteworthy that PCB 97 was found in all adult plasma samples and was the second highest concentration congener. Concentrations of PCBs have been extensively studied in birds of the North American Great Lakes region [15]. PCB residues and toxic effects have been observed in different bird species [9,15]. Mean concentrations of total PCB in the plasma of Great Lakes were slightly greater than the mean total PCB concentrations in plasma of the BFAL on Midway Atoll, but considerably greater than those in Laysan albatrosses. Mean PCB concentrations in plasma of black-footed chicks were less than those in Great Lakes-influenced, but greater than those in interior bald eagle chicks [1,15].

The TEQs in the BFAL adults were considerably greater than those of the chicks. The BFAL may be at risk for PCB toxicological effects, and the concentrations of these toxic congeners would be expected to increase over the lifetime of the BFAL. PCBs 77, 81, and 126 have the highest TEFs for birds because all these PCB congeners contain non-*ortho* chlorine atoms and chlorine substituents at the *meta-para* carbon atoms, making these congeners more likely to be accumulated rather than metabolized [8–10].

### PCB concentrations in the preen oil of adults

**Fig 5:**
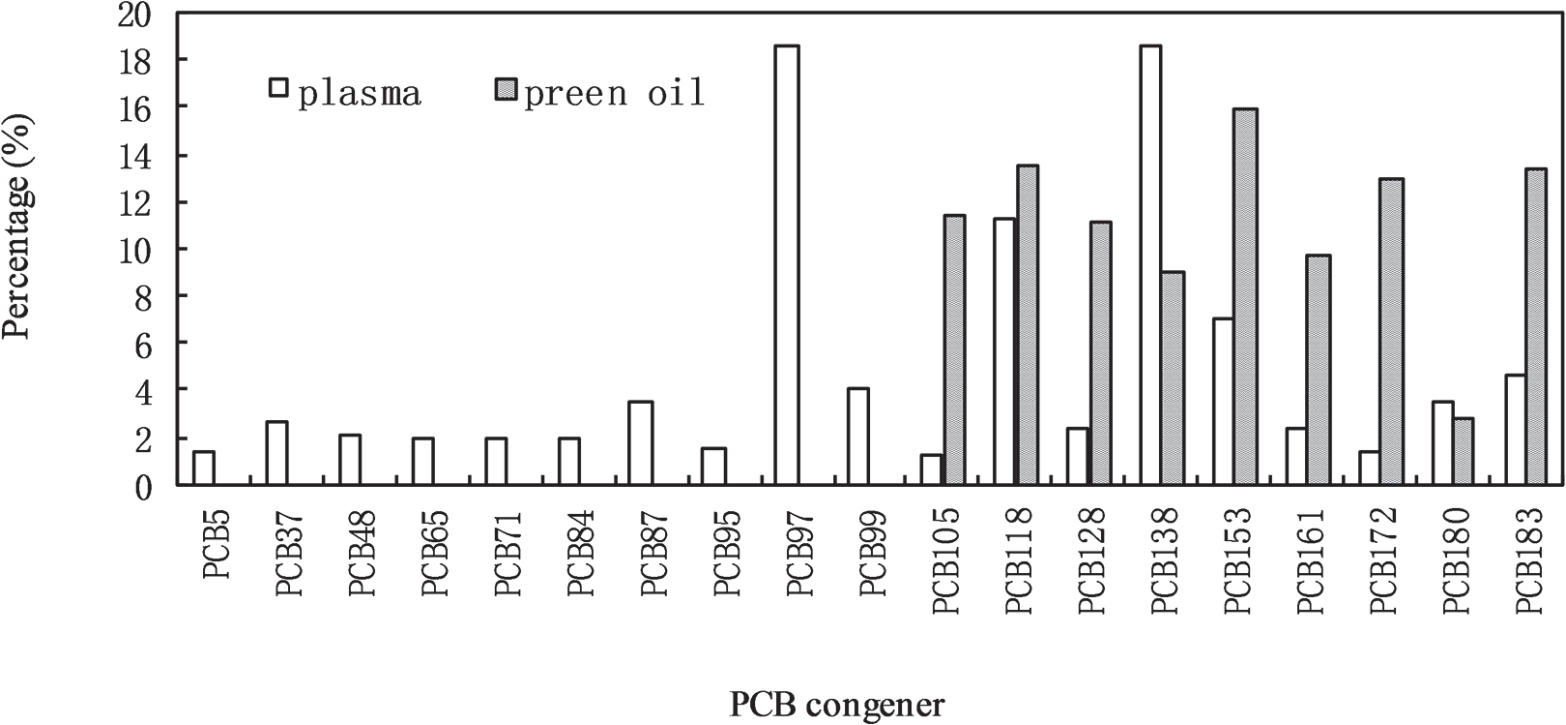
Percentage (%) of individual PCB congener to total PCB concentration in the plasma and preen oil samples of the adult individuals from Midway Atoll, North Pacific Ocean.

Fig 2B shows the concentrations of total PCBs in the preen oil samples of BFAL adults. The concentrations of the total PCBs in the preen oil samples of adults ranged from 1693 to 39404 (mean 10122) ng g^-1^ ww collected in B8 and bulky dump sampling sites. The major congeners in the preen oil of adult samples included PCBs 105, 118, 128, 138, 153, 161, 172, and 183, accounting for 97% of the total PCBs (Fig 5). The average concentrations of total PCBs in the adult preen oil samples (1693–39404 ng g^-1^ ww) were approximately 74–78 times higher than those in the adult plasma samples (22.8–504.5 ng g^-1^ ww). The total PCB concentrations in these adult preen oil samples from the B8 sampling site were similar to those from the bulky dump sampling site. It appears that the total PCB concentrations in the preen oil samples were inversely related to the total body masses of BFAL adults (*p* < 0.0001) (Fig 4C). A slight inverse correlation was observed between the total PCB concentrations in the plasma samples and those in the preen oil samples (*p* < 0.0001) (Fig 4D). The PCBs detected in the preen oil samples were mainly highly chlorinated congeners, whereas PCB congeners at various chlorination levels were detected in the plasma samples (Fig 5).

### PCB exposure to BFAL chicks and adults in the Midway Atoll

BFAL range in the North Pacific Ocean where PCBs were detected [1,8,18,19]. BFAL commonly scavenge plastics [20–22]. Plastic ingestion is another route of PCB exposure to BFAL. Specific-feeding habits of BFAL may also influence its organochlorine accumulation. Guruge et al. [21,22] found high ratios of PCDDs/PCDFs to PCBs in the Midway Atoll albatrosses and suggested multiple sources of pollution in the area. The albatrosses that nest on Midway Atoll are exposed to non-point pollution sources and local pollution sources [1,21,22]. Air samples collected south of Midway Atoll contained a mean total PCB concentration of 130 pg m^-3^, and samples of seawater taken along this route in the North Pacific Ocean showed a mean total concentration of 24 pg m^-3^.

Moreover, the position in the food chain exposes albatross to PCBs, and this determines the overall congener profile. Differences in congener distributions between trophic levels is dependent on natural “weathering” and the sorting of compounds by solubility, volatility and stability [13–15]. The congener profiles in seabirds are dependent on a variety of factors: foraging range, food preferences, behavior, metabolic capabilities, species lipid contents, depuration rates, uptake of certain congeners through diet, biotransformation. In migratory seabirds, the concentrations of PCBs are a function of time and space. PCB exposure at one location may differ from another location [12,13,20]. Fish-eating birds tend to have a predominance of higher chlorinated congeners. In general, the proportions of some higher chlorinated congeners (≥ 5 Cl) increase through the food chain, while the lower chlorinated PCB congeners make up a smaller percentage of the congeners on the top of the food chain [12,23]. The amount of lower chlorinated congeners decreases as the trophic level increases, but atmospheric transport of PCBs tends to lower chlorinated congeners [1]. The congener profiles were unique in the BFAL from Midway Atoll because of the low abundance of the lower chlorinated PCB congeners (< 5 Cl atoms). PCBs 138, 153, 170, 180, and 187 are the most common congeners found in the highest abundance in biota samples. These congeners contain chlorine atoms in positions 2,4,5-, 2,3,4- or 2,3,5-positions in one or both of the phenyl rings, *para* positions on both rings, and the absence of adjacent unsubstituted *meta* and *para* positions [13,19,24–26]. PCBs 138, 153, 170, 180, and 183 are the major components of Aroclors 1254 and 1260 [24]. PCBs with no adjacent chlorines in the *meta-para* locations are metabolized to the greatest degree (PCBs 101, 110, 141, 149, 174), followed by *meta-ortho* locations (PCBs 60+56, 66, 74, 99, 105, 118, 128, 137, 138, 170+190, 171, 177) [20–22]. PCB congeners with no adjacent unsubstituted positions are metabolized very slowly or not at all (PCBs 146, 153+132, 172+197, 178, 180, 182+187, 183, 194, 200+157, 201, 203+196) [20–22]. PCBs 105, 118, 128, 138, 153, 161, 172, and 183 accounted for 97% of the total PCBs detected in the preen oil from the BFAL adults on Midway Atoll in the present study.

PCB degradation in birds constantly exposed to high levels of PCBs is faster because the enzymes in seabird body involved in metabolism are induced to a greater extent [27,28]. PCB congeners with no *meta-para* chlorines can be metabolized by the phenobarbital-type enzymes that are responsible for the metabolism of PCBs in birds [21,22]. This makes the highly toxic *ortho-meta* chlorine unsubstituted congeners more likely to be accumulated [20–22]. Generally, birds do not have the ability to metabolize the highly chlorinated PCBs. However, albatross may have some xenobiotic metabolizing enzyme activities that other bird species may not [1].

Jones et al. [1] reported that the ratio of PCB 101 to PCB 99 was approximately 1:10 in the albatross tissue samples from Midway Atoll. The predominance of PCB 99 over PCB 101 may be due to differential metabolism in BFAL [1]. PCB 101 contains adjacent unsubstituted *meta* and *para* positions, which are required for oxidative metabolism and elimination. It has been shown that PCB 101 can be metabolized by fish-eating sea birds [24,25]. PCB 99 has chlorine substituents at both *para* positions and, therefore, does not have unsubstituted adjacent *meta* and *para* positions, making this congener more likely to be accumulated. The average PCB 101: PCB 99 ratio in the plasma of BFAL chicks and adults is close to 1:20 in the present study. These results suggest that the chicks and adults can accumulate PCB 99 to a greater extent relative to PCB 101.

Phillips et al. [28] compared the chick metabolic rate and growth of three species of albatross. The seabirds grow slowly and accumulate large lipid reserves during the nestling stage. The chicks peak in their body mass during the late rearing period, but lose mass prior to fledging. The chick feeding varies because of an unpredictable food supply from the parents. In their early development stage, the chicks were building up their fat reserves. The older BFAL chicks were losing some weight prior to flying. The adults were larger, but have less fat than the chicks. Phillips et al. [28] showed that the total PCB concentrations and total PCB body burdens in the adults were higher than those in the chicks, but not proportional to the increase in the mass of the BFAL adults. Guruge et al. [21] observed a dilution of total PCBs during the rapid growth stage in nestlings. Lemmetyinen et al. [29] looked at the PCB levels at different life stages of artic terns and herring gulls and found the highest levels in the adults and the newly hatched chicks; and in both species, the chicks showed a sharp decrease in total PCB concentrations in 2–4 weeks, with the herring gulls decreasing even more prior to fledging. There was a general increase in PCB concentrations as getting older observed with the common cormorants from Japan [30].

White-tailed eagles that contained high PCB concentrations were emaciated and contained a low fat content in the breast muscle [13]. Due to dilution, the PCB levels in a lighter weight chick tend to be higher than those in heavier-weight chicks [29,31]. PCB exposure will increase in times of starvation when lipids are mobilized, and PCBs are redistributed from adipose tissue to muscle and then from muscle to adipose tissue after feeding [32]. The smaller chicks may have been abandoned in a state of starvation and, therefore, their PCB levels would be higher due to lipid utilization and slow PCB metabolism and secretion [32]. Food supply is one of the main factors in the growth rate of chicks. The seabird chicks show patterns of infrequent weight gains from feedings and weight loss due to metabolism between feedings [28,33].

During the incubation and guarding periods, adult BFAL on Midway are in a state of fasting and lose approximately 25% of their body mass [1,34]. Therefore, the PCB levels in the adults during those periods would tend to be high. During the nesting period, the adult albatross feed 300 to 500 miles north of the Hawaiian Islands chain at the major oceanic currents, not near Midway [1]. The adult BFAL are not accumulating organochlorines from a local source of contamination (from Midway Atoll itself), and the contamination seen in the adult BFAL is representative of the more general contamination of the North Pacific [1,22]. The food supplied to the chicks by their parents would be representative of this general contamination of the North Pacific. However, prior to fledging, the young albatross chicks spend their entire time on the ground at Midway Atoll, and during this period of time, the chicks are susceptible to local sources of PCB contamination on the atoll. Nestling juveniles provide the best indicators for local environment contamination because they are stationary during this time and would be most affected by their surrounding environment [21,22].

In addition to non-point sources, evidence of the present study and comparison to similar studies suggest that the BFAL on Midway Atoll might be exposed to local pollution sources. The PCB concentrations in the BFAL chick plasma samples varied within 97-fold (2.3–223.8 ng g^-1^ ww), whereas the PCB concentrations in the BFAL adult plasma samples varied within 22-fold (22.8 to 504.5 ng g^-1^ ww). The large PCB concentration differences among the individual chicks relative to the adults suggest that some of the chicks may be exposed to high levels of PCBs around their nests, i.e., local pollution sources. The low abundance of the low chlorinated PCB congeners (<5 Cl atoms) in the BFAL samples also suggests local pollution sources as the atmospheric transport of PCBs tends to be lower chlorinated congeners.

## Conclusions

The analysis of individual congeners makes the calculation of concentrations of potential toxic congeners possible as well as individual congener distribution within individual plasma and preen oil samples and gives a more representative measurement of the exposure of the birds to PCBs. Total PCB concentrations were in the ng g^-1^ and μg g^-1^ ww concentration ranges in the plasma and preen oil samples, respectively, of the BFAL chicks and adults on Midway Atoll. Local PCB pollution is on Midway Atoll.
